# Analysis of Falls Efficacy Scale and Vulnerable Elders Survey as Predictors of Falls

**DOI:** 10.7759/cureus.14471

**Published:** 2021-04-13

**Authors:** Marna Greenberg, Jeanne Jacoby, Robert D Barraco, Ali R Yazdanyar, Ryan M Surmaitis, Alexander Youngdahl, Richard B Chow, Sofia M Murillo, Allen H Zeng, Bryan G Kane

**Affiliations:** 1 Department of Emergency and Hospital Medicine, Lehigh Valley Health Network, Allentown, USA; 2 Department of Surgery, Lehigh Valley Health Network, Allentown, USA

**Keywords:** elderly falls, fes, ves, emergency department

## Abstract

Introduction

Falls are the leading cause of injury-related death among older adults according to the Centers for Disease Control and Prevention (CDC). The Falls Efficacy Scale (FES) and Vulnerable Elder Survey (VES-13) are validated screening tools used to assess concern of falling, health deterioration and functional decline. We set out to determine if the FES or VES-13 could serve as a predictor of falls among older adults in the Emergency Department (ED) setting.

Methods

This prospective pilot cohort study was conducted at a Level 1 Trauma Center. ED patients aged ≥65 were eligible for the study if they had a mechanical fall risk defined by CDC criteria. After consent and enrollment, FES and the VES surveys were completed. Participants were followed by phone quarterly, and results of the one-year follow-up self-report of fall history described.

Results

There were 200 subjects enrolled and after excluding those that were withdrawn, deceased, or lost to follow-up, 184 were available for analysis of their follow-up visit at 12 months. A greater proportion of the participants were women (108 (58.7%) vs 76 (41.3%); P=0.88). The average age of the study participants was 74.2±7.3 years. There was no significant difference in age between men and women (median: 73 vs 73; p=0.47).

At the follow-up visit, 33 (17.9%) had a reported fall. The mean age did not significantly differ when comparing those with versus without a fall (75.6 vs 73.9; p=0.24). There was no significant difference in the proportion with a VES-13 ≥ 3 when comparing those with and without a reported fall (45.5% vs 37.8%; p = 0.41). The median FES score did not differ among those with as compared to without a fall (11 vs 10; p=0.12).

Conclusions

Subjects who had a VES-13 score of ≥3 were statistically no more likely to have fallen than those with a score of <3. Additionally, the FES score did not statistically differ when comparing those who had fallen to those who had not. Further research into alternative screening methods in the ED setting for fall risk is recommended.

## Introduction

The Centers for Disease Control and Prevention (CDC) reports that each year, there are millions of adults 65 years and older who suffer a fall. Moreover, older adults are at increased risk for subsequent falls and fall-associated mortality after an index fall [1-4]. Falls have been shown to result in a decline in function both from the trauma and decreased confidence in the ability to perform functional activities [5]. In 2014, approximately 2.8 million older adults were treated in Emergency Departments (ED) for fall-related injuries, of which 800,000 were later hospitalized [6]. In addition to the mortality associated with falls, the total economic burden was approximately $50 billion in 2015 [7].

There are numerous gender-specific risk factors that are associated with falls; in one of the first studies to assess these differences in older adults, non-fatal fall-related injuries disproportionately affected the health of older women, with an almost twofold increase in fracture rate among women [8]. More recently, a cross-sectional study looked at the prevalence of falls and associated sex-specific risk factors [9]. The study concluded that lifestyle and behavior may differ by sex, which may explain varying underlying health conditions [9]. In women, higher odds of falling were associated with nutritional risk, polypharmacy, alcohol consumption, and osteoporosis. Interestingly, in men, a higher level of education was found to be a protective factor against falls [9].

Because of the number of older adults at risk, there is an ever-increasing demand for screening and interventions that reduce fall risk and functional decline. The Falls Efficacy Scale (FES) is a tool that assesses fall-related self-efficacy and fear of falling, which may lead to a decline in physical fitness and an increase in fall risk due to physical frailty [10]. In addition to the FES, the Vulnerable Elder Survey (VES-13) is used to predict the functional impairment of older adults and identify individuals at higher risk of falls. Through assessing Activities of Daily Living (ADLs) and Instrumental ADLs (IADLs) within the survey, this tool may provide more information to assist in predicting mortality and future hospitalization [11]. The CDC has also designed the Stopping Elderly Accidents, Deaths & Injuries (STEADI) Tool Kit for health care providers to help assess elderly patients’ fall risk [12]. These can all potentially be used in conjunction to lower fall risk in the older adult population [12].

There have been several studies that have examined the relationship between the FES or VES-13 and functional decline and falls. One study used VES-13 as a predictor for long-term decline and mortality in community-dwelling older adults and found that the VES-13 is an excellent predictor of functional decline and survivability over five years [9]. The implications of this study were that it provided useful prognostic information that could help determine the utility and aggressiveness of preventative or therapeutic interventions.

Despite the existing literature on the topic, few studies have involved ED-specific interventions which may assist clinicians to prevent functional decline and falls in older aged adults. Moreover, there is a lack of published literature on the use of the FES or VES-13 in the ED setting to identify persons at risk of falling [13]. In this study, we sought to determine the effectiveness of the FES and the VES-13 in predicting falls in older adults as well as to determine if differences exist in preferences and outcomes between women and men. 

## Materials and methods

This pilot study was conducted with a prospective cohort design. The setting was an ED at a Level 1 Trauma Center in Northeast Pennsylvania with approximately 90,000 annual visits across all age groups. Patients presenting to the ED were screened for study eligibility with the goal of recruiting 200 participants. Subjects were considered eligible if they had a mechanical fall risk as defined by CDC criteria [14]: had fallen in the past year, reported worry about falling, or admitted to feeling unsteady when standing or walking. Additional inclusion criteria were: age ≥ 65, English-speaking, able to provide consent to participate in the study and able to be discharged home from the ED.

All study subjects completed the baseline FES and VES-13 and the participant’s risk for falling was discussed. Subsequently, a standardized simple questionnaire (Appendix 1) regarding fall history was conducted at 6-weeks, 3 months, 6 months, 9 months, and 12-months follow-up intervals from baseline. Subjects who were not reached by telephone were mailed (certified) the follow-up surveys and were requested to complete the surveys and mail them back for analysis. The VES-13 and FES are described elsewhere [10, 11]. According to the VES-13 tool, scores that were ≥ 3 were considered positive screens for a high risk of falls [11]. The FES tool has a 4-point scale ranging from “not at all concerned” to “very concerned” used to rate concern for falling when performing activities of daily living such as taking a shower or bath or going up or downstairs. The overall score of the FES was determined by adding all the individual question scores together and the scores would range from 7 (no concern about falling) to 28 (severe concern about falling).

The information from the data collection forms and surveys was stored in a secure database in preparation for analysis. All data collected was de-identified to protect patient privacy, and access was granted only to the necessary research team members to collect, input, or analyze the data. All study participants provided informed consent. This study was approved by the Institutional Review Board. 

Descriptive statistics were calculated for continuous and categorical variables. Categorical variables are presented and frequencies (percentages). Associations between categorical variables were calculated using the Chi-squared test. Continuous variables are presented as means (±standard deviations) and medians (2nd Quartile, 3rd Quartile). The distribution of continuous variables was assessed both graphically and by the Shapiro-Wilk test of normality. Continuous variables were described using either parametric or nonparametric methods as appropriate. Two-sample Wilcoxon Rank Sum test was used to assess for a difference across a two-level categorical variable for a continuous variable when the assumptions for a two-sample t-test were not met. Spearman’s rank correlation and Spearman’s partial rank-order correlation was used to assess the correlation between VES-13 and FES scores without and with accounting for age and gender, respectively. 

Logistic regression was used to assess the association between the occurrence of a fall at 12-months with VES-13, age, and gender. For the logistic models, the VES-13 scores were analyzed as a binary covariate dichotomized at a value of 3. The Area Under the Curve (AUC) and the Receiver Operating Characteristic (ROC) curve were used to characterize the performance of each model in classifying a fall. The classification performance was assessed and compared by screening tools (VES-13 vs. FES) and gender (men vs. women). 

## Results

**Figure 1:**
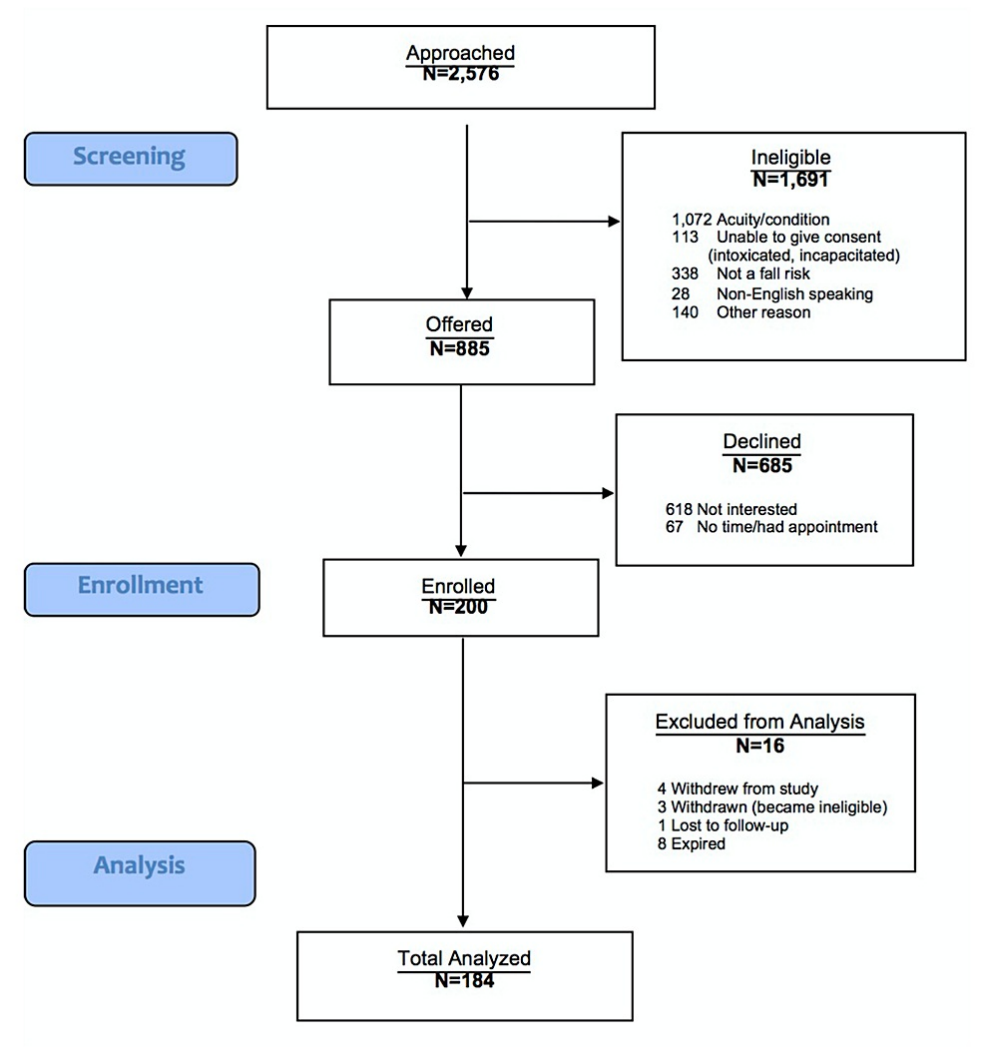
CONSORT Flow Diagram. Schematic of study population. CONSORT: CONsolidated Standards Of Reporting Trials

Two-thousand-five-hundred-seventy-six subjects in the emergency department were screened for inclusion in the study; of those 1691 were ineligible to participate (1072 due to the severity of their illness, 113 did not have the capacity to consent, 338 did not have a fall risk, 28 did not speak English, and 140 for other reasons). Another 685 were not eligible because they declined to participate. The remaining 200 subjects were initially enrolled in the study. After exclusions, including those who withdrew (n = 4), were deemed ineligible (n = 3), deceased (n = 8), or were lost to follow-up (n = 1), a sample size of 184 remained at the 12-month follow-up visit (Figure 1). The average age of the study participants was 74.2±7.3 years. There was no significant difference in age between men and women (median: 73 vs 73; p=0.47). A greater proportion of the participants were women (108 (58.7%) vs 76 (41.3%); p=0.88). One-hundred-seventy-four (94.6%) subjects identified as Caucasian, 4 (2.2%) identified as Black/African American, 1 (0.5%) identified as American Indian/Alaskan Native, 1 (0.5%), and 4 (2.2%) as other. Five (2.7%) of the subjects identified as Hispanic or Latino and the remainder did not. All of the subjects recruited lived at home and were discharged to their home prior environment.

The median FES and VES-13 scores were 10 (Interquartile Range (IQR): 8, 13) and 2 (IQR:1,4), respectively. The median FES score was 9 (IQR: 7,12) and 10 (IQR: 8,13) in men and women, respectively. The FES scores for men (median: 9 (IQR: 7,12)) and women (median: 10 ;IQR: 8,13)) were not significantly different (p=0.21). The VES-13 scores for men (median: 2 (IQR: 1,4)) and women (median:2 ;IQR: 1,3.5)) were not significantly different (p=0.85). 

The proportion with a VES-13 score ≥ 3 did not differ between men and women (18.6% vs 23.6%; p=0.86). The correlation between VES-13 and FES score was 0.62 (p<0.01). The partial correlation between VES-13 and FES accounting for age and gender was 0.63 (p<0.01). There were 33 (17.9%) participants who reported a fall at the 12-month follow-up. Age did not significantly differ when comparing those with and without a fall (median: 74.0 vs 72.0; p=0.17). There was no significant difference in the proportion of falls reported by men as compared to women (18.4% vs 17.6%; p=0.89).

There was no significant difference in the proportion with a VES-13 ≥ 3 when comparing those with and without a reported fall (45.5% vs 37.8%; p=0.41). The median FES score did not differ among those with as compared to without a fall (11 vs 10; p=0.12).

The odds of a reported fall at 12-months, with and without accounting for the other potential confounding factors, is shown in Table 1. Based on the logistic regression model, the predicted risk of a fall increased from 14.4% to 32% as the VES-13 score increased from 0 to 10. The predicted risk of a fall as the VES-13 score increased from 0 to 10 remained stable at approximately 18% for men; however, in women, it increased from 12.3% to 40.4%. This interaction between gender and VES-13 did not reach statistical significance (p=0.27).

**Table 1:** Association between Age, Gender, and VES-13 with the Odds of a Fall OR, Odds Ratio; CI, Confidence Interval; yr, year; VES, Vulnerable Elder Survey

	Unadjusted OR	Adjusted OR
	OR [95% CI]	OR [95% CI]
VES-13 (3 or higher)	1.37 [0.64,2.94]	1.13 [0.46,2.72]
Age (per yr.)	1.03 [0.98,1.08]	1.03 [0.97,1.09]
Female	0.95 [0.44,2.03]	0.97 [0.45,2.09]

**Figure 2:**
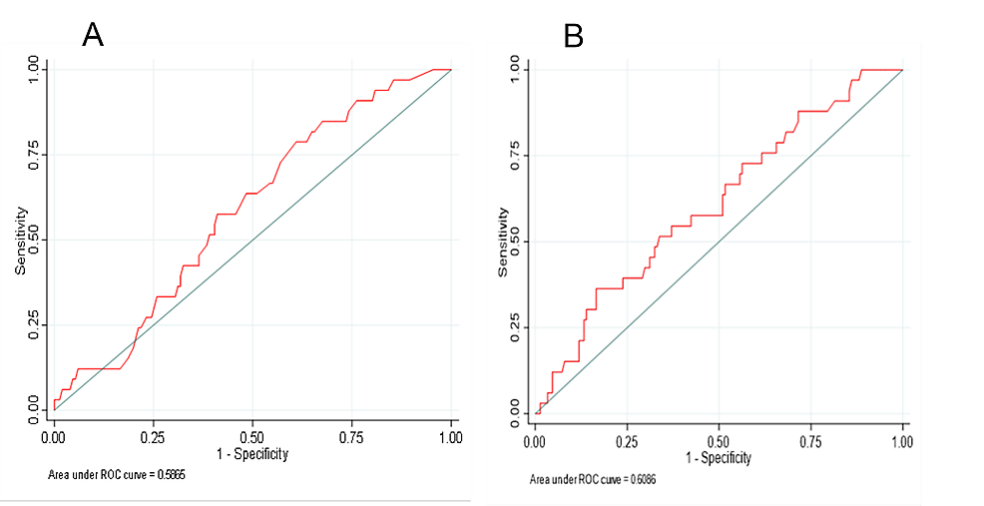
Model Predicting a fall by 12-months using (A) VES-13 (≥3) or (B) Total FES score (A) ROC for VES-13 (≥3); (B) ROC for total FES score

Figures 2A, 2B display the ROC curves for models using VES-13 and FES, respectively. The AUC was 0.587 for a model including VES-13 and 0.609 for the model including FES. There was no significant difference in AUC when comparing VES-13 and FES (p=0.28). 

The AUC of the gender-stratified model including VES-13 was 0.4615 for men and 0.5385 for women (p=0.42) while models including FES score had an AUC of 0.4129 for men and 0.5871 for women (p=0.12).

**Figure 3:**
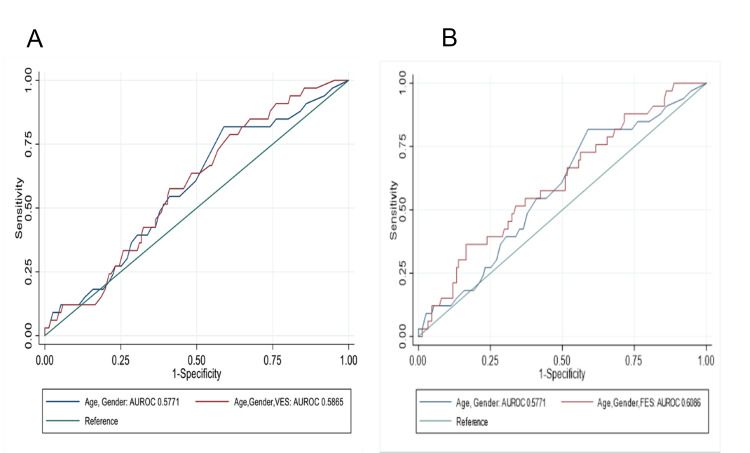
Prediction of a fall by 12-months with age- and gender-adjusted model with and without (A) VES-13 (≥3) or (B) Total FES score

Figures 3A, 3B display the incremental change in the AUC with the addition of VES-13 or FES to the model containing age and gender. The addition of VES-13 to a model including age and sex did not significantly improve the model (AUC, 0.5771 vs. 0.5865; p=0.52). Similarly, the addition of FES to a model which included age and sex did not significantly improve the model (AUC, 0.5771 vs. 0.6086; p=0.54).

## Discussion

In this cohort of older adults visiting an ED, nearly one-fifth suffered a fall by 1 year. We found that both VES-13 and the FES scores performed poorly in their ability to predict a fall at 12 months. The VES-13 and FES did not significantly differ in their ability to classify a patient at risk for a fall at 1 year. Moreover, the addition of either VES-13 or FES to patient characteristics of age and gender was not associated with a significant improvement in the ability to predict a fall in 1 year. 

Previous studies have investigated the relationship between the VES-13 and FES as predictors for functional status or mortality. Sandlund et al. studied functional status and survival outcomes in elderly patients as predicted by the VES-13 [15]. The prediction outcomes in that study were over 5 years and the outcomes demonstrated a linear relationship between scores on the VES-13 and the odds of death and functional decline [15]. In the current study, we sought to use both the VES-13 and FES as screening tools to attempt to find out if they were useful predictors of risk, and have not found a strong correlation. Previously, falls efficacy, as measured by the Modified Falls Efficacy Scale (MFES), and postural balance were used to predict fall outcomes [16]. Unfortunately, no significant relationship between MFES and fall risk was found, but interestingly they found an association between MFES and future gait limitations [16]. In our analysis, it appears that the FES also does not have a clear association with fall risk, but we did not look at other outcomes that could contribute to falls such as worsening gait.

Some of the most significant impacts of investigating screening tools for fall risk are the benefits of cost reduction from reduced rates of hospitalization due to falls and improved healthcare quality provided to the rapidly growing geriatric population [17]. It may be useful to study other screening tools in order to make adjustments for new screening methods. The objective is to find ways to reduce the number of falls after the discharge of ED patients. If a good screening tool were found to accurately predict fall, the tool could be incorporated into clinical pathways for all patients aged ≥ 65 and in a higher risk category for falls as determined by CDC criteria [14].

Future research can be done to investigate other screening tools or strategies to more accurately identify those at risk for falls so that they can receive targeted interventions. By accurately predicting those at greatest risk for falls, better interventions and management options could be implemented to prevent falls and associated functional decline. In addition, further investigation of predictive risk factors and comorbidities that lead to falls may provide even more information into screening and interventions to prevent falls. Further research into gender differences in the selection of fall prevention interventions and fall outcomes may help develop a more patient-centered ED decision tool for fall prevention.

Limitations

This study has several limitations. The study may lack generalizability in that it is a single-site study that included only patients who spoke English and were to be discharged home from the ED. The impact on the study findings due to the high rate of screen failure, which resulted in less than 10% enrollment of those approached, is unknown. Any predictive tool for geriatric falls screening must account for the multi-factorial nature of fall risk. Once screened, how the results are utilized will further impact the predictive nature of the screen. The screening itself may provide something akin to a Hawthorne effect on the patient or those who care for the patient and become aware of the fact a screen was performed [18]. Utilizing the screening to trigger interventions further confounds the predictive ability of the screen.

## Conclusions

There were some sex-specific differences in the rate of falls. However, the FES screening tool did not statistically differ when comparing those who did fall to those who did not. In addition, an increased VES-13 score ≥ 3 did not correspond to increased likelihood of falls compared to a VES-13 score < 3. Further research into alternative screening methods in the ED setting for fall risk is recommended. 
